# A strategic framework for pathology faculty onboarding

**DOI:** 10.1016/j.acpath.2026.100293

**Published:** 2026-09-23

**Authors:** Aparna Mahajan, Karen Kaul, Sara E. Wobker, Liron Pantanowitz, Anna Prilutska, Dorina Gui

**Affiliations:** a Department of Surgical Pathology and Laboratory Medicine, University of Wisconsin School of Medicine and Public Health, University of Wisconsin–Madison, Madison, WI, USA; b Department of Pathology and Laboratory Medicine, Endeavor Health, Evanston, IL, USA; c Department of Pathology and Laboratory Medicine, University of North Carolina School of Medicine, Chapel Hill, NC, USA; d Department of Pathology, University of Pittsburgh School of Medicine, Pittsburgh, PA, USA; e College of Health Professions and Sciences, University of Wisconsin–Milwaukee, Milwaukee, WI, USA; f Department of Pathology and Laboratory Medicine, University of California, Davis School of Medicine, Sacramento, CA, USA

**Keywords:** Engagement, Job satisfaction, Manual, Onboarding, Pathology, Retention

## Abstract

Effective oversight of talent acquisition, integration, management, and retention in an academic pathology department is critical to sustaining its tripartite mission of clinical service, research, and education. Despite substantial focus on these areas, the crucial initial transition and onboarding period for new faculty is often treated as an afterthought. Substantial evidence shows that well-structured onboarding programs boost productivity, satisfaction, and retention. Yet, a 2024 Association for Advancing Physician and Provider Recruitment report found that just over half of physicians and clinicians receive formal onboarding. Without it, new faculty often face a “fog of war” that delays process adoption, risks inconsistency in patient-care practices, and heightens professional isolation. Recent shifts in academic pathology—the pandemic-driven “great resignation,” the rise of multilocation subspecialty settings, and hybrid work environments—have highlighted the limits of traditional quick-start guides. These changes demand a recalibrated, phase-based approach that balances cognitive load, capitalizes on early heightened receptivity, and nurtures sustained engagement. To address this, we propose a seven-pillar framework: Administrative Foundations; Systems and Technical Training; Role Clarity and Expectations; Institutional and Cultural Orientation; Networking and Integration; Faculty Engagement and Support; and Professional and Personal Well-Being. Sequenced delivery of information—from prearrival digital dashboards to 30-day performance reviews and life assistance—creates a learner-centered roadmap that eliminates logistical hurdles, builds technical fluency, clarifies roles, fosters relationships, and supports personal transitions. Shifting from transactional checklists to a structured, long-term onboarding model, departments can accelerate new faculty competence, deepen alignment with departmental values, and strengthen retention, sustaining excellence in an ever-changing healthcare landscape.

## The strategic imperative of talent management

For academic leaders, the strategic oversight of its talent acquisition, integration, management, and retention is a critical responsibility that directly underpins their department's tripartite mission – clinical service, research, and education. Despite the significant resources and attention allocated by pathology departments to attracting and retaining top faculty talent – evidenced through assigning faculty roles such as the Chair of the Recruitment Committee, or the Vice Chair of Faculty Development – the crucial initial transition and onboarding period for new hires is frequently an afterthought. Substantial evidence supports the value of robust and well-structured new-hire onboarding programs. Organizations that invest in this area consistently report a range of tangible benefits that directly contribute to both individual and organizational success. Data suggests that structured onboarding leads to increased productivity, satisfaction, and retention within healthcare systems. It also provides an opportunity to promote departmental culture, incorporating one-on-one meetings with important stakeholders early in the onboarding process.^1^, ^2^, ^3^, ^4^, ^5^, ^6^, ^7^, ^8^, ^9^

A 2024 onboarding research report by the Association for Advancing Physician and Provider Recruitment highlighted a strong connection between the presence of a structured onboarding program and job satisfaction. However, the same report indicated that just over half of the physicians and clinicians reported receiving a formal onboarding experience, with the rest receiving some informal support or none at all.^10^ In essence, while a good onboarding process drives new faculty job satisfaction, these programs are not yet ubiquitous. Without a structured onboarding system, it is inevitable that new faculty feel “left to their own devices,” which can lead to slow adoption of the new processes, a lack of consistency in patient care approaches (especially in subspecialized settings), and a feeling of isolation from both pathologist and clinician colleagues.

Sharing novel approaches to onboarding can help institutions understand the needs of new faculty and develop robust, structured processes that can be tailored to specific institutional needs. This opportunity was discussed extensively in 2024 at the Clinical Service Leaders Committee meeting of the Association of Academic Pathology, which spurred the development of this article. The central inquiry was to establish the foundational principles for a robust onboarding program and was accompanied by a discussion of best practices across pathology departments recognized for their robust onboarding programs—including the University of Wisconsin–Madison, University of Pittsburgh, University of California–Davis, University of North Carolina, and Northshore.

## Shifts in the pathology landscape

In the last few years, academic pathology has undergone significant shifts that have fundamentally reshaped the way we do our work.

1. The great resignation – A 2022 CHG Healthcare survey of 500 physicians indicated that 43% of physicians changed jobs and an additional 8% retired during the first two years of the COVID-19 pandemic.^11^ Other reports published concurrently also indicated elevated attrition or strong physician intent to leave their current practice.^12^^,^^13^
2. Pathology was not immune to these trends. The departure of seasoned faculty eliminates opportunities for organic, sustained transfer of institutional knowledge. This knowledge, including established relationships with clinical colleagues and historical context informing departmental decisions, would have naturally unfolded over an extended period of time.
3. Multi-location multi-institution subspecialty practice settings – Contemporary pathology increasingly operates across multiple physical locations and across multiple institutions in a highly subspecialized model. While subspecialization offers numerous benefits—such as improved diagnostic accuracy, enhanced education and research, and higher faculty satisfaction—it also demands increased staffing to cover all specialized areas. Additionally, these new settings driven by subspecialized guidelines and precision medicine, necessitate structured knowledge transfer—representing a shift from traditional, observation-based knowledge transfer mechanisms.
4. Hybrid work environment – While explicit data on the prevalence of remote/hybrid work models are limited, anecdotal evidence suggests a substantial increase from prepandemic levels. This shift to working from home (WFH), supported by advances in digital and telepathology and increasing use of artificial intelligence (AI), offers flexibility, cost savings, improved work-life balance, wider access to talent, and systemic efficiency. Unfortunately, it also raises barriers to traditional forms of knowledge and culture transfer and the process of relationship building. The informal learning that occurs through hallway conversations and ad hoc consultations is significantly diminished in these environments. Additionally, WFH introduces technology, regulatory, teaching, and security challenges.

These shifts necessitate a re-evaluation and recalibration of our approach to onboarding our new hires. The traditional ‘quick-start guide’ approach to onboarding, which brings together different aspects into a brief initial sprint, is no longer adequate.

## Redefining onboarding for future preparedness

During the onboarding process, the organization often has all the necessary information or can readily obtain it from the new hire. Conversely, new hires generally begin their roles with significant information deficits and are frequently unaware of what critical information they are missing. This “fog of war” leads to anxiety, stress, and reduced efficiency and can delay faculty from performing their jobs – e.g., they cannot sign out cases if their hospital privileges are not approved or they have not been trained on the Laboratory Information System (LIS). In such an environment, an onboarding approach characterized by one-off orientations, scattered emails, and opportunistic introductions, compels newcomers to piece together vital information through trial and error. This challenge is further exacerbated by the fact that resources, processes, physical spaces, and culture differ significantly between institutions, and even within a multi-institution practice**.** A unique paradox emerges – new hires are overwhelmed with information, yet they struggle to build and connect the specific knowledge crucial for integration, job success, and career progression.

An effective onboarding program demands structure in its breadth and scope, as well as in the timing and cadence of its delivery. It must provide the right information, support, and guidance at the right moment. Delivering excessive information too early can result in extraneous cognitive overload, with the risk of degrading retention. Conversely, providing too little information too late means new faculty miss the benefits of their “honeymoon period,” a time characterized by heightened enthusiasm, receptiveness, goodwill from colleagues, and fewer responsibilities as they ramp up. The onboarding program must carefully balance the needs and expectations of the department with a delivery cadence that maximizes the benefits to the new hire. Additionally, it must not be seen only as a discrete short-term event to merely get the new hire to commence their immediate work. Rather, it must aim to get them fully “on-board” into the department's lifeblood, setting them on a path to sustained engagement and career growth.^14^^,^^15^

## The seven pillars of onboarding success

Based on recurring themes observed across the aforementioned programs, this seven-pillar, phase-based framework offers a comprehensive, learner-centered roadmap that scaffolds new hires through the things they need to hit the ground running, feel supported, and build toward long-term success (Fig. 1).

**Fig. 1:**
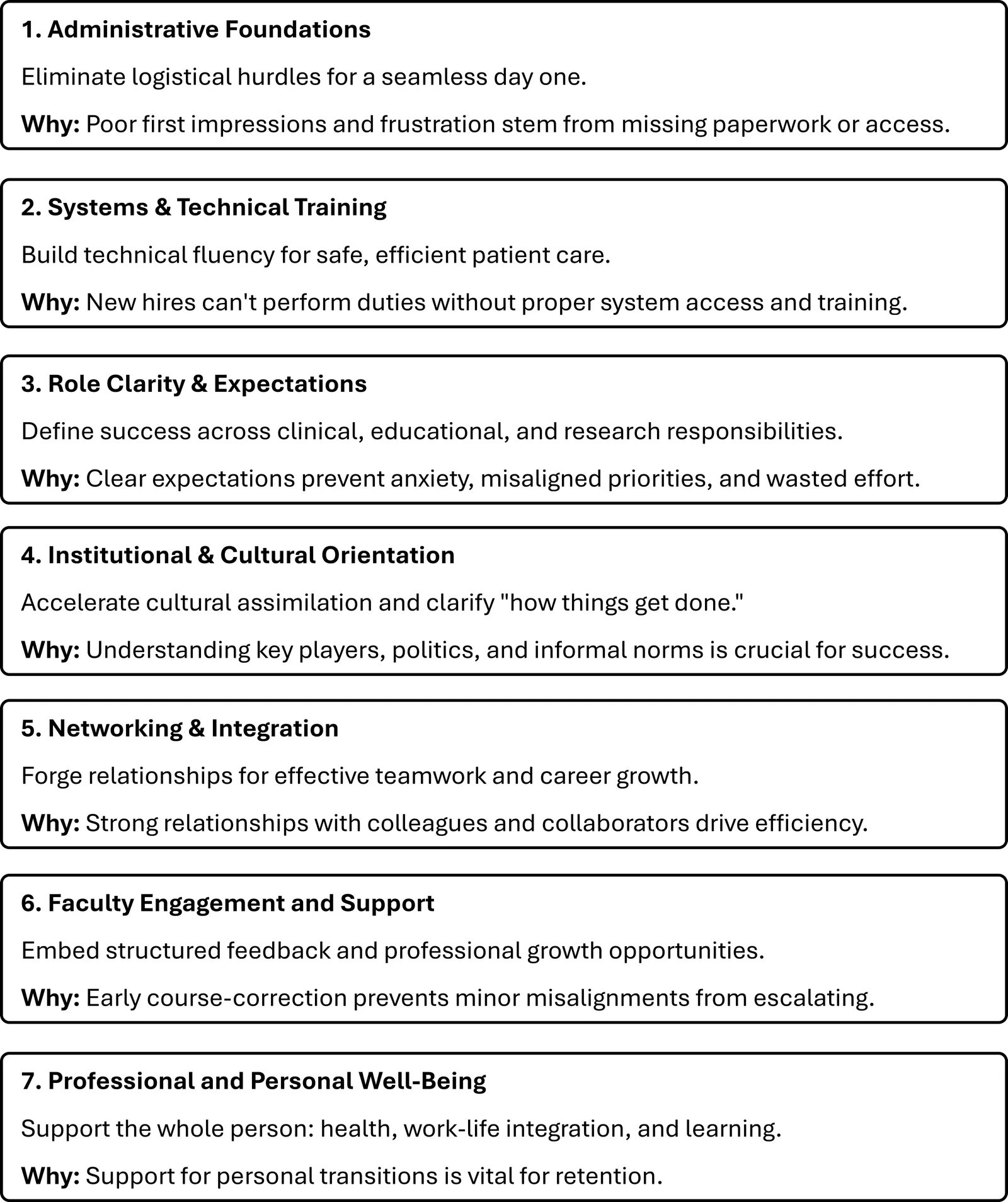
The seven pillars of effective onboarding.

### Administrative foundations

Observations from our benchmarking exercise indicated that beginning roughly two to four weeks before the official start date, new hires received some access to their new department’s “Onboarding Dashboard,” a centralized digital checklist that tracks both universal and program-specific tasks. Universally, hires completed I-9 verification (for identity and eligibility for US employment), background checks, hospital privilege and malpractice insurance applications, direct-deposit setup (for electronic transfer of paychecks into a bank account), benefits and retirement enrollment, and disclosure-of-outside-interests. Program-specific items—such as Institutional Review Board (IRB) credentialing for research-intensive roles, and radiation-safety permits for certain subspecialties—were appended by local administrators. On arrival, in some departments, a dedicated coordinator met the new hire to issue their photo ID, activate parking and building access, assign office and lab keys, set up voicemail and network credentials, and distribute an up-to-date departmental directory. Over the first three days, the coordinator finalized payroll and leave-reporting enrollment, activated professional expense accounts, confirmed emergency contact information, and tracked outstanding specialty-credentialing tasks—ideally visible in the dashboard so both the hire and their supervisor could monitor completion in real time.

### Systems and technical training

To ensure new hires could immediately engage with clinical, education, and/or research workflows, mandatory e-learning modules Electronic Medical Record (EMR)/LIS fundamentals, Health Insurance Portability and Accountability Act (HIPAA), data-privacy, fire and life safety, and critical-incident management) in some cases were assigned pre-arrival. In the early days on campus, IT specialists imaged and configured their workstations, installed, validated, and provided one-on-one coaching on dictation and voice-recognition software (e.g., Dragon Medical and Winscribe), and demonstrated digital-pathology platforms including gross-room cameras, microscope-mounted cameras, and whole slide scanners.

Throughout this crucial period, departments also provided robust digital resources. This included making dashboards or worklists readily available within the LIS that centralized all helpful links for areas like send-out tests, electronic policies, external laboratory contacts, library resources, and departmental websites. Beyond digital access, a structured clinical practice manual encompassing subspecialty guidelines for clinical sign-out specific to that institution or even subspecialty, on-call guidelines, and helpful resources for laboratory and pathology support (such as helpdesk contacts, in-basket chart help, new faculty orientation manuals (Fig. 2), and multisite phone numbers)—all consolidated in one place–helped new hires get up to speed with institutional standards and requirements at clinical sign-out.

**Fig. 2:**
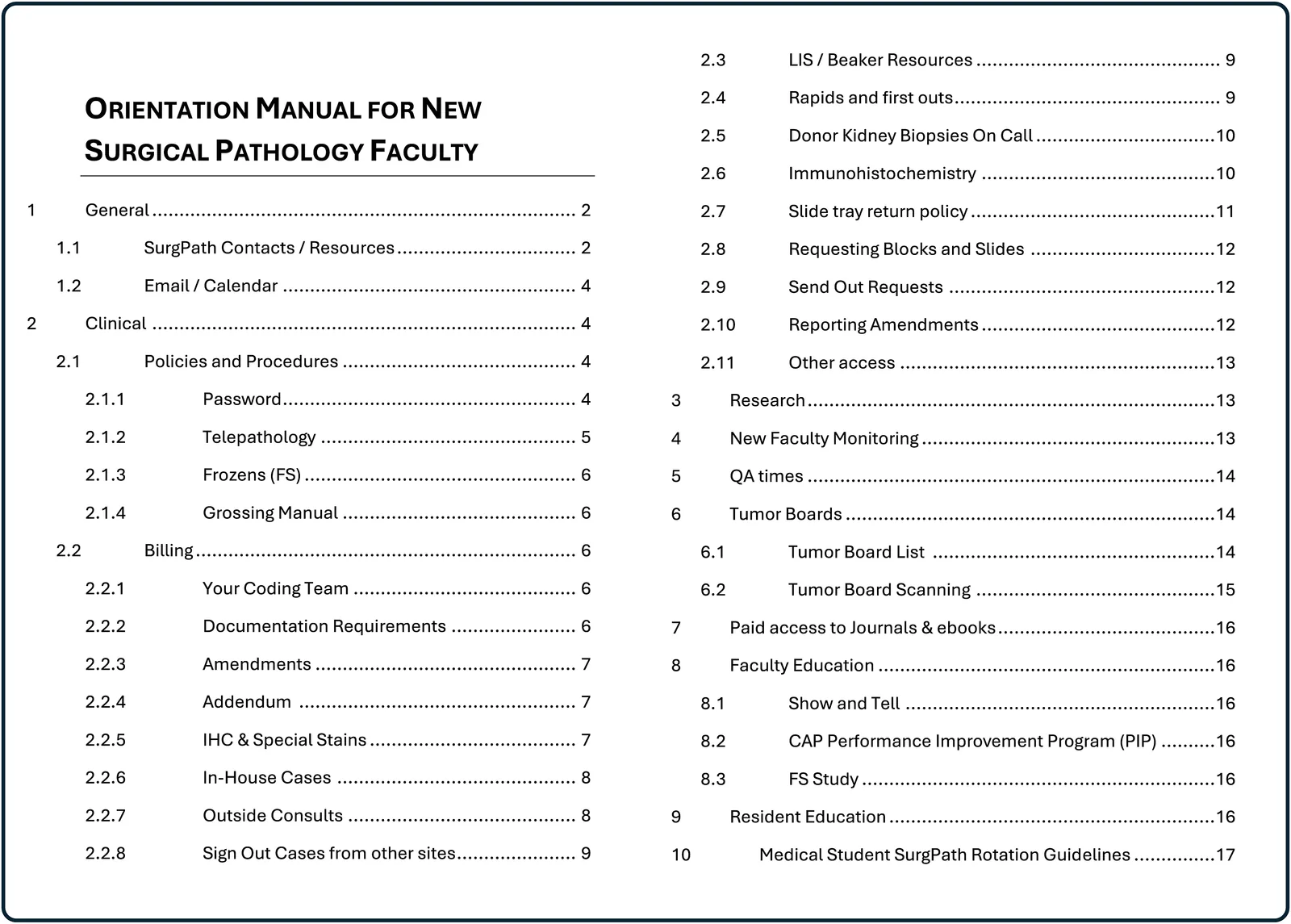
Table of contents: orientation manual for new surgical pathology faculty at the University of Wisconsin-Madison.

Over the ensuing early weeks of employment, new hires could observe routine sign-out sessions handled by other faculty, participate in tumor boards, and gradually assume their own case interpretation. Throughout this period, a designated EMR “physician-champion” at some centers met individually to tailor user-interface elements—smart-phrases, order-sets, and personalized sign-out dashboards—to the pathologist's subspecialty and case mix. Programs with teaching or research tracks may layer on additional sessions, such as virtual-microscopy tutorials, research-informatics drop-in clinics, or pedagogy tool orientations for educators.

### Role clarity and expectations

Prior to setting foot on campus, some organizations provided draft clinical and teaching schedules alongside anticipated service coverage targets, case-volume benchmarks, and teaching loads—complete with proposed committee assignments and grant submission deadlines. In the first week, a formal orientation meeting with division leadership finalized service weeks (including multisite coverage rotations), on-call calendars, laboratory management responsibilities, and formal committee or task-force roles. A comprehensive onboarding manual spanning detailed clinical protocols, billing workflows, immunohistochemistry and molecular pathology standard operating procedures (SOPs), quality-assurance metrics, and research grant budgeting templates provided the new hire with transparent performance benchmarks and criteria for independent practice. For faculty with robust academic or research expectations, the onboarding manual could also potentially integrate teaching evaluation rubrics, mentorship agreement templates, promotion-and-tenure guidelines, grant-related administration, and sample academic year planning calendars to set clear short- and long-term goals. Once the initial surge of onboarding cadence slows down, specifically walking new hires through the LIS-driven dashboards, clinical service manuals, and quality assurance (QA) processes can be crucial.

### Institutional and cultural orientation

The new hire was given structured, guided tours in the first week to introduce them to their new work environment, such as the anatomic pathology suite, clinical labs, research cores, and the medical school campus; brief “office hours” sessions with the Anatomic Pathology Director, Chief Administrative Officer, Surgical Pathology Director, and assigned mentor clarified reporting structures and strategic priorities. During weeks 2–4, formal meetings with departmental and operational leaders delved into departmental history, mission, and values; QA and peer-review (including focused and ongoing professional practice evaluation processes), morbidity and mortality conferences, and patient-safety huddles, illustrating how metrics like turnaround time drive daily operations. Select programs enhanced this with discussions on equity and inclusion efforts and community engagement workshops.

### Networking and integration

In the early days of a new hire's appointment, the department Chair and the leader for faculty development met with the incoming faculty member and played an active role in selecting the mentoring and peer-support team, ensuring all departmental components of onboarding were in place. During the first two weeks, each new hire is paired with both a formal mentor—a senior faculty member within their subspecialty—and an informal “buddy,” a peer who helped with day-to-day questions and facilitated social and professional introductions. A sequence of “speed-networking” meetings connected them with key collaborators: clinical providers such as surgical and medical oncologists, radiologists, QA program leads, IT analysts, research administrative officers, and lab technician teams. Subspecialty and service line directors (e.g., frozen section and telepathology) hosted small group meetings to explore collaborative research and clinical service opportunities. Some institutions layered on social network orientations—briefings on faculty interest groups, decision-making committees, and informal social events to help new faculty navigate the department's unwritten cultural norms and locate internal champions.

### Faculty engagement and support

At a formal 30-day check-in, the new hire met with their division director and assigned mentor to review progress with onboarding as well as progress against clinical service benchmarks, initial case volumes, teaching effectiveness, and early research milestones—surfacing any misalignments and co-authoring an individualized development plan to ensure alignment with departmental expectations.

### Professional and personal well-being

Recognizing that new faculty often face simultaneous personal transitions—finding new housing, securing suitable schools for their children, and establishing a social network—some departments embedded “life-settling” support where possible. New hires received curated tips and personal experiences on housing options, school district evaluations, spousal employment leads, and cultural integration resources. This included neighborhood overviews, guidance on evaluating school options, contacts for local employers known to hire academic spouses, and links to faculty-led community groups. Some departments additionally invited the new hire's family and children to these events. By proactively easing these off-duty challenges, the department ensured faculty could devote their full energy to professional responsibilities from day one.

## Conclusion

The evolving academic pathology landscape demands a fundamentally reimagined approach to faculty hiring, integration, and retention. To address this need, academic medical centers should improve their onboarding process for newly hired faculty. There are benefits from successful onboarding for both the pathology department and the new employee. For the organization, onboarding accelerates the learning curve for new faculty, enabling faster productivity, helping them feel welcomed and thus more likely to stay long-term, strengthening engagement and their alignment with the department's values and culture, as well as reducing liability related to potential errors. This also improves the department's reputation as a good place to work. For the new employee, onboarding provides them with clarity about their role and expectations, as well as how their success will be measured. Furthermore, this creates a sense of belonging, empowers them early on, makes them feel more valued, reduces anxiety, and helps them build the supportive network they will need with coworkers, mentors, and leadership.

A successful onboarding program needs to move beyond a transactional “checklist” mindset and instead must deliver information, resources, and relationship-building opportunities to get the new hire on a path to sustained growth. By structuring the process into chronological phases and sustaining support through long-term development, departments can minimize cognitive overload, accelerate cultural assimilation, and foster early alignment on expectations. Creating a formal onboarding manual is encouraged. It is equally important to keep this manual updated by continually surveying newly hired faculty to solicit their feedback.

The proposed seven-pillar framework—Administrative Foundations; Systems and Technical Training; Role Clarity and Expectations; Institutional and Cultural Orientation; Networking and Integration; Faculty Engagement and Support; and Professional and Personal Well-Being—provides a learner-centered roadmap that supports new hires through their onboarding process. Combined, these pillars streamline logistics, build skills, clarify expectations, strengthen relationships, and support smooth transitions—driving satisfaction, retention, and performance. As academic pathology departments strive to sustain excellence in clinical service, research, and education, embracing this comprehensive, phase-based onboarding model will not only smooth each new hire's transition but also strengthen the collective capacity of the team and organization to adapt and thrive in an ever-changing healthcare landscape.

## Declaration of competing interest

The authors declare no conflicts of interest related to this manuscript.
